# Autistic adults’ experiences of managing wellbeing and implications for social prescribing

**DOI:** 10.1080/09687599.2023.2263628

**Published:** 2023-10-12

**Authors:** Charlotte Featherstone, Richard Sharpe, Nick Axford, Sheena Asthana, Kerryn Husk

**Affiliations:** a NIHR Applied Research Collaboration South West Peninsula, NIHR PenARC, Peninsula Medical School, Plymouth, England; b Public Health, Cornwall County Council, Truro, Cornwall, England; c University of Exeter Medical School, Penryn, Cornwall, England; d NIHR PenARC, NIHR Applied Research Collaboration South West Peninsula, Plymouth, England; e Peninsula Medical School, University of Plymouth, Plymouth, England

**Keywords:** Autism, wellbeing, self-determination, social prescribing

## Abstract

Autistic people demonstrate poor outcomes on objective measures of wellbeing, yet research centring lived experience provides a more nuanced picture. There is growing support for person-centred, holistic and community approaches to enhancing wellbeing for autistic people. Social prescribing may be one such approach. This qualitative study explored the concept of wellbeing for autistic adults – including barriers and self-management – and the implications of this for modifying social prescribing. It involved semi-structured interviews with 21 autistic adults in the UK. Reflexive thematic analysis of the data supports research suggesting that self-determination may underlie many aspects of wellbeing for autistic people. The COVID-19 pandemic provided new opportunities to develop wellbeing strategies but also had negative impacts. Social prescribing could promote self-determination by signposting autistic people to peer support opportunities building on intrinsic interests.

## Points of interest

- This interview-based study asked autistic adults to define wellbeing in their own words.
- The ability to pursue personal wellbeing goals and interests encouraged feelings of competence, connection, acceptance and a positive identity.
- Social and environmental factors, as well as autistic traits, could have both positive and negative impacts on wellbeing.
- Receiving an autism diagnosis and accessing support promoted rather than diminished independence and choice, suggesting a need for support should not be considered a poor outcome for autistic people and an accurate diagnosis at any age can be helpful
- Social prescribing approaches, which connect people to community-based supports, could assist autistic adults with finding and approaching supportive communities and building on interests to enhance wellbeing.

## Introduction

A population’s wellbeing can be an important indicator of broader quality of life, used to compare outcomes across regions and drive policy decisions (Office for National Statistics, 2018). Although definitions of wellbeing vary, most models comprise multiple dimensions. For example, a general population survey conducted by the United Kingdom’s Office for National Statistics (ONS) found the most acceptable definition of wellbeing comprises intrapersonal domains (e.g. life satisfaction and physical and mental health), interpersonal factors (e.g. relationships and loneliness), employment, leisure activities, living standards, and wider factors such as the economy and environment (Office for National Statistics 2016). Some dimensions can be measured objectively, while others rely on subjective evaluation (Department of Health 2014). Collective factors can also impact on wellbeing. These include shared values and interests of a population (such as location), relationships, networks, shared learning, social cohesiveness, equity and social justice, which interact with individual factors to achieve an overall sense of community wellbeing (Coburn and Gormally 2020; Atkinson et al. 2017; Rayner et al. 2018).

Autistic adults are known to experience poor outcomes in many areas pertaining to wellbeing, including high rates of physical and mental health problems, unemployment, isolation, educational disadvantage and financial hardship (Brugha et al. 2011; Croen et al. 2015; Howlin 2021). Studies measuring quality of life in autistic adults have found low scores across many domains when compared to population norms or non-autistic controls (Holmes et al. 2020; Lawson et al. 2020) and negative correlations between autistic traits and quality of life (Oakley et al. 2021). However, Oakley et al. (2021) observed a high level of individual variability, especially for adults, warranting further interrogation of subjective quality of life, including protective factors and developed coping strategies. Findings informed by lived experiences of autistic people, typically using qualitative methods, also present a more nuanced narrative (Welch et al. 2022; Welch et al. 2019). They sometimes reframe medical diagnostic criteria and functional differences considered maladaptive such as how self-stimulatory behaviour may aid emotion regulation (Kapp et al. 2019) and have elicited concepts shared in autism-related communities but not classified in diagnostic criteria, including meltdowns, inertia, burnout and masking. These concepts are linked to wellbeing, impacting mental health, social interactions and daily activities (Bradley et al. 2021; Buckle et al. 2021; Cassidy et al. 2018; Cage and Troxell-Whitman 2019; Hull et al. 2017; Raymaker et al. 2020; Welch et al. 2021). A coproduced quality of life scale for autistic samples led to inclusion of nine autism-specific items including autistic identity, sensory processing and healthcare barriers (McConachie et al. 2018).

Critical perspectives argue that measures developed in the general population, despite methodological robustness, may apply normative, individualistic and medicalised standards to autism research (Jones 2022; Lam, Sabnis, Valcarlos, et al. 2021; Mason et al. 2021). This leads to poor outcomes being attributed to autism and resulting in a prevailing assumption that being autistic is incompatible with thriving (Chapman and Carel 2022). Interventions often target individual behaviours and traits, yet these have shown little collective success over time at reducing wellbeing disparities (Howlin 2021). Wellbeing-related constructs are less emphasised in outcome measurement (Featherstone et al. 2022). However, the constructs identified through subjective accounts emphasise contextual factors such as access to services, features of environments and social pressures on wellbeing, supporting models such as the neurodiversity paradigm and social model of disability which propose that poor fit between individual conditions (in this case, the lived reality of autism) and social context lead to disability (Shakespeare 2013).

A literature review on self-reported outcomes found self-determination may instead underlie many areas pertaining to wellbeing for autistic people including employment, healthcare access and social participation (Kim 2019). Self-determination theory (SDT) describes a spectrum of motivation for action ranging from intrinsic motivation to external drivers, with higher levels of intrinsic motivation being more indicative of self-determination (Ryan and Deci 2000). Three psychological needs – autonomy, connectedness and competence – contribute to intrinsic motivation within SDT (Gagne & Deci, 2005). Higher self-determination has been found to relate to life satisfaction in autistic young adults (White, Flanagan, and Nadig 2018), and that markers assumed to indicate good outcomes, such as independent living, are not always indicative of life satisfaction and positive overall wellbeing (Mason et al. 2021; Scheeren et al. 2022; Pellicano et al. 2022; Taylor and Henninger 2015). Mason et al. (2021) argue defining successful outcomes should be based on assessing each individual’s values. These findings indicate a need for wellbeing support for autistic adults which takes self-determination, individual differences and social context into account (Kim 2019). Other recommendations for improving support for autistic adults include using flexible and person-centred approaches which identify existing strengths to build skills and social connections, and allowing lived experience to drive wider research and practice (Howlin 2021; Murray, Lesser, and Lawson 2005).

Social prescribing describes a healthcare model where health professionals connect people to wellbeing-enhancing activity in communities, such as exercise groups, nature referrals and advice services (Bickerdike et al. 2017; Chatterjee et al. 2018; Kimberlee 2015; Polley et al. 2017). Social prescribing is often positioned as countering, complementing or extending traditional medical approaches, as a tailored and person-centred model taking account of biopsychosocial factors including individual values, goals and barriers (Calderón-Larrañaga et al. 2022; Ogden 2018). It often targets people managing chronic illness or experiencing socially-derived problems such as loneliness, where medical intervention is insufficient to address wellbeing (Polley et al. 2017). The typical social prescribing model involves a link worker to coproduce an appropriate social prescription, matching clients’ goals with the link worker’s knowledge of community networks (Polley et al. 2017). This role can be crucial to successful social prescribing (Hazeldine et al. 2021), as is the voluntary, community and social enterprise (VCSE) sector which provides much of the end-point support (Polley et al. 2017).

Findings on social prescribing’s effectiveness are mixed, with quantitative studies often demonstrating poor methodological quality, low uptake, and inconsistent outcome measures (Blodgett, Kaushal, and Harkness 2022; Bickerdike et al. 2017). However, many features of social prescribing, such as person-centred tailoring and collaborative approaches, may facilitate engagement with community-based supports for autistic adults, although current social prescribing models may require some adaptation to benefit autistic people in the referral pathway (Featherstone et al. 2022). This might include widening referral methods and offering flexible levels of social interaction, as well as increasing understanding of autism amongst link workers, for whom a lack of training around ‘complex’ patients is often perceived as a barrier to success (Hazeldine et al. 2021; Holding et al. 2020; Wildman et al. 2019). Furthermore, qualitative research has linked social prescribing to self-determination through mechanisms of a supportive link worker relationship, centring of individuals’ interests and goals, community participation and enabling self-management of health and wellbeing; these increased intrinsic motivation around health management and enhanced social competence (Bhatti et al. 2021; Hanlon et al. 2021).

However, few primary research studies have investigated whether social prescribing may benefit autistic people (Charlton et al. 2020; Featherstone et al. 2022). As part of a broader research project aiming to understand how social prescribing could be tailored towards this group, we formulated this study to explore the concept of wellbeing for autistic adults and the implications for modifying social prescribing. The study was conducted during the first year of the Covid-19 pandemic in the United Kingdom, which affected wellbeing across populations and sectors, negatively impacting mental health, social isolation and finances, issues which were more pronounced for disabled people (Office for National Statistics 2020a; Office for National Statistics 2020b; Emerson et al. 2022). Impacts for autistic people were mixed, due to differing individual experiences of the effects on daily routines, social demands and access to services (Bundy et al. 2022; Pais and Knapp 2021). The pandemic also impacted the social prescribing pathway, highlighting benefits and disadvantages of social prescribing models within this context and resulting in changes to practice likely to persist post-pandemic (Westlake et al. 2022). We aimed to understand how individuals defined wellbeing, their strategies for managing wellbeing and experiences of barriers to meeting their wellbeing needs, within the context of Covid-19.

## Research process

### Theoretical approach

Our study adopted a critical realist philosophy, which bridges constructivist and positivist approaches by recognising multiple interpretations and perspectives on reality. This is pertinent to the debates on how typical standards have been applied to autistic people without always considering diverse lived experience, which is often framed as an issue of heterogeneity in positivist approaches that aim to generate robust and standardised definitions and interventions for autism (Botha 2021; Chapman and Veit 2021; Woods et al. 2018; Kourti 2021). While a wholly constructivist approach may risk undermining observable differences generated by medical research, as well as the distinct social disadvantages affecting autistic people (Botha 2021), critical realism gives room for alternative models such as the neurodiversity paradigm and social model of disability. These positions represent those led by scholars with lived experience. Applying critical realism allows for the co-existence of paradigms and methods used to construct knowledge, in this case about subjective wellbeing; this can highlight power differentials and contextual factors in how the mechanisms of reality are realised (Rosqvist et al. 2022). For example, the “double empathy” theory re-evaluates what biomedical models have reinforced as theory of mind deficits in autism by presenting evidence of bi-directional difficulties and contextual factors affecting perspective-taking (Milton 2012), with a negative impact on social and emotional wellbeing for autistic people.

We applied reflexive thematic analysis to analyse interviews, as this is a suitable methodology for the critical realist ontology (Braun and Clarke 2022), which requires reflection on how knowledge is produced (Botha 2021; Kourti 2021).

### Sampling strategy

All but one participant had been recruited to a previous survey by the researchers that investigated autistic adults’ wellbeing and access to healthcare and communities during Covid-19. Those who had consented to be contacted about future research opportunities were invited to take part in this study. Original recruitment methods involved advertising the study through the Autistica Discover Network, charities and organisations providing local support for autistic adults, university disability services, local governmental autism partnership boards, social media and autism-related forums. One participant in the interview study was recruited by word of mouth. Participants could either have a formal autism diagnosis or self-identify as autistic (ensuring representation of those who were either not able to access, or preferred not to seek, a formal diagnosis).

### Ethics

All procedures were approved by the University of Plymouth’s Faculty of Health Ethics Board (reference: 19/20-1311). Participants received an information sheet and consent form in advance, which included their right to withdraw their data at any time prior to analysis. Their consent to the procedures was audio recorded along with their full names, except for one participant who gave written consent due to participating *via* email. Following interviews, participants received a debrief form with details of wellbeing support and advice.

Adjustments made at a universal level to support a diverse group of autistic individuals to participate included sending participants the interview questions in advance to reduce uncertainty and facilitate communication. Participants were also given options for taking part using Zoom, either as a video call, by phone (supported by Zoom) or by text using Zoom’s chat function. All participants who self-selected for the study informed the researchers if further adjustments to procedures were required. These included providing easy-read information about interview concepts, such as social prescribing, to facilitate participation for those with learning disabilities, and allowing the use of email to communicate, considered an appropriate accessible method of data collection for interviews with autistic people (Nicolaidis et al. 2019); an ethical amendment was granted to accommodate this. Use of terminology adhered to community guidelines and preferences (Bottema-Beutel et al. 2021). In transcription we removed names of people, organisations and places that might identify participants or others.

### Data collection

Online interviews took place in summer 2021, so both interviewer (CF) and participants took part remotely. Interviews used a semi-structured format, with participants asked initially if there was any particular area of wellbeing they wanted to begin with. The interview schedule (Appendix 1) began by focusing on what wellbeing meant to each individual, with questions including: ‘What does wellbeing mean to you?’ and ‘What does it mean to feel at your best?’ Participants were asked about their strategies for keeping physically and mentally well, how these were developed, and when these had helped or been less helpful. They were also asked if the Covid-19 pandemic had affected how they were able to keep well. The complete interview covered a range of topics, including access to healthcare, places and environments, views on nature-based social prescriptions, and questions on community belonging, including the impact of the pandemic. In this paper we focus on responses relating to wellbeing experiences.

We used Zoom to conduct interviews, record audio files and generate transcripts. The auto-generated transcripts were corrected jointly by a researcher (CF) and a professional transcription service. Transcripts contained spoken dialogue only, in line with the approach of thematic analysis. The process of correcting transcripts enabled familiarisation with their content.

### Data analysis

In accordance with procedures for reflexive thematic analysis, one researcher (CF) coded transcripts inductively, using colour coding to differentiate responses for each research question. Initial codes were entered into a spreadsheet and organised into preliminary categories. We used NVivo to begin organising initial themes. Possible themes and links to theory were discussed in meetings with the broader research team, leading to further revisions to the overall theme structure.

## Results

### Participants

Participants (*N* = 21) were adults over 18 years old living in the UK. They were not asked if they had a formal autism diagnosis or self-identified, but all participants who had taken part in the previous survey had scored above the threshold of ≥6 on the AQ-10 measure, suggesting they would meet criteria for an autism assessment (Allison, Auyeung, and Baron-Cohen 2012), and one participant recruited later had a formal autism diagnosis. 11 participants were male, 7 female and 3 nonbinary. 18 participants identified their ethnic background as white (British or other) and one as Black Caribbean; 2 did not state their ethnicity. Additional disabilities reported by participants, which may influence their experiences of wellbeing, included mental health conditions (e.g. anxiety, depression, schizophrenia) (8 participants), physical disability or long-term illness (e.g. heart condition, cerebral palsy, chronic fatigue) (7 participants), specific learning difficulties (e.g. dyslexia, dyspraxia) (5 participants), ADHD (5 participants) and intellectual disabilities (2 participants). Age, measured in bands, ranged from under 25 to over 66, with most participants falling in the bracket of 56-65.

#### Personal wellbeing

This theme comprised intra-personal factors involved in wellbeing. The first subtheme identifies ways of defining wellbeing and factors influencing subjective understanding. The second subtheme identifies participants’ strategies and actions for managing wellbeing and their development. The third subtheme identifies how intrinsic traits and interests contributed to wellbeing and achievement of goals. The fourth subtheme reveals barriers internal to the person identified as affecting wellbeing.

##### “My normal is very different”: wellbeing as multidimensional

Most participants viewed wellbeing as a multifaceted concept beyond simply physical or mental health to include such concepts as: environment; social connections; spirituality; finances; a sense of control; the ability to perform daily activities; achieving goals. Some understandings were subjective and had been developed through personal experience, learnt values and attitudes towards health. For example, education influenced personal definitions of wellbeing.

“I think ever since I did some study in philosophy I’ve had a very holistic approach towards, you know, what constitutes the human being, human society - you’re both individual and community” (George, M, 66+)

Other participants referred to recognised models, such as “five ways to wellbeing”. Some participants perceived that their understanding might defy accepted ideas of wellbeing that they inferred from the phrasing of interview questions.

“Normal is a very broad term […] my normal is very different from other people’s normal wellbeing” (Gavin, M, 46-55)

Other participants avoided comparisons with standards, favouring internal feelings of self-acceptance and contentment as wellbeing markers. Many accounts portrayed a need for harmony and equilibrium.

“Mental and physical health, and the two together. Not one. So if you’re not well with one of them, your overall wellbeing is not good. So you have to be well in both of them to have good wellbeing.” (Alan, M, 56-65)

Some participants contrasted wellbeing to their current state, for example having more energy, being free of pain and being happier. This suggested they did not regard wellbeing as a realistic goal for themselves currently.

“For me to feel my best is to just not feel tired, not just, just not feel permanently exhausted, and I do […] I am never, never at 100%; I’m always at 50 or running below 50 the entire time.” (Lauren, F, 36-45)

##### “I Wonder if I should actually just get some running shoes and start running”: wellbeing through actions and routines

Following on from identifying which areas of wellbeing were important to them, participants described actions and routines they used to help maintain those. Actions towards promoting physical health included exercise (running, swimming, sports, walking and gardening) and diet, but some participants also noted benefits to their mental health by taking part in exercise, and most people did not exercise for physical benefit alone. Other strategies to enhance emotional wellbeing included maintaining a sense of predictability and control over life, such as keeping to a regular routine or working from home. Some participants had learnt further techniques to manage emotional wellbeing through therapy, while others felt they had an innate pragmatic thinking style which helped with processing problems; some attributed this to being autistic.

Many participants adapted their environment to manage sensory input and also sought out positive environments to improve their wellbeing. Natural environments were preferred by many participants, as these had lower sensory input and calming features, which encouraged mindfulness and reflection.

“I quite like being beside a river or brook, by running water, so the noise of it, it helps me calm myself down, it’s something to focus on” (Noel, M, 46-55)

Accounts also revealed how participants had developed their actions and strategies - through trial and error, education (for example, a post-cancer course) and seizing opportunities.

“I used to run away a lot, because sometimes the anxiety would get so overwhelming I couldn’t cope […] I started thinking, I wonder if I should actually just get some running shoes and start running. And I did, and now I have a like a section in the day, where I just actually run […] provided that happens, I’m fine.” (Fiona, F, 46-55)

Small actions, such as purchasing inexpensive equipment, were noted for how quickly these could facilitate routines and contribute to broader wellbeing outcomes, for instance being able to access nature for wellbeing.

“If you told me before I bought the map that […] it would make such a difference I wouldn’t have believed you […] it would have helped way back when I was really having problems” (Alan, M, 56-65).

Some participants looked to external validation, such as research on the benefits of exercise, to justify their strategies. Some also identified as a ‘healthy person’, prioritising exercise, diet, and preferring not to consume alcohol, smoke or use medication excessively.

Actions and routines participants used to support wellbeing were frequently linked to a sense of enjoyment, and there was less discussion of longer-term health goals. However, routines could sometimes become restrictive.

“The biggest thing for me is a stable, solid, rigid routine […] If anything gets in the way of me doing that […] I feel like I can’t cope.” (Lauren, F, 36-45)

##### “You’ve done something for yourself”: interests, achievements and personal strengths

Autistic people commonly have focused interests, categorised under restrictive and stereotyped behaviours in diagnostic criteria (Buckley 2017). Participants in the present study described interests and occupations including art, reading, running, computer games, driving, metalwork, martial arts, theatre, learning, archaeology, nature, DJing, music and writing Some participants led successful careers based on their interests, while others engaged in hobbies, voluntary work or more casual interests and daily activities, but these nevertheless provided occupation, stimulation and a sense of purpose. Some accounts suggested absorption in engaging with interests helped distract from daily stressors.

“It’s a sort of a place where I hyper-concentrate and I don’t notice anything else, whilst I’m painting, and I just paint for hours and hours […] I don’t really hear, see or think or do anything other than what I’m doing.” (Fiona, F, 46-55)

Interests could also provide opportunities to connect with others, either directly or in a more abstract sense. For example, one participant felt her interest in theatre helped her to “observe humanity” (Cheryl, F, 56-65), while another’s interest in archaeology gave him “emotional connectivity” (Ivan, M, 56-65) to a historical context, that he could convey to visitors of an exhibit he volunteered at. Another participant described how he connected with others through writing.

“You’re not just writing it for yourself […] you have to make sure it resonates with everyone without them having to ask you what you meant” (Anton, M, 26-35).

Developing skills and abilities through engaging with interests also gave participants a sense of achievement and success.

“I literally planted my, my garden and I like doing very physical stuff sometimes because… it sort of helps to lift my mood you know […] it’s going to be amazing looking you know, and you just got a sense of pride, you have physically achieved something” (Noel, M, 46-55)

Participants also identified personality strengths that they felt resulted from their autistic traits, including attention to detail, perseverance and creativity. Having enhanced sensory experiences led participants to feel more absorbed in their interests. Some considered these traits beneficial for their career choices, such as law and art.

##### “It just seems so boring”: internal barriers to wellbeing

Participants described how intrinsic traits and health problems could sometimes hinder achievement of wellbeing goals. Mental health issues such as depression, anxiety and trauma affected daily activities, relationships and enjoyment of environments, and several participants had experienced suicidal thoughts and suicide attempts. Some described physical health conditions, such as chronic pain and fatigue, limiting exercise, work and daily living. Other co-occurring conditions such as ADHD and dyspraxia could also cause barriers. Some participants experienced low motivation towards maintaining their wellbeing.

“I do nothing and it’s terrible, I used to go jogging once a day last year, but I’ve completely lost the motivation to exercise […] It just seems so boring” (Alex, NB, 26-35).

For some, executive dysfunction impacted on their motivation and ability to organise their daily routines, such as remembering to take medication. Some also felt a permanent sense of burnout and overwhelm.

“Just getting up and existing - for me, it’s just like I can get up and within 15-20 minutes of getting up I’m exhausted, again, I feel like I need to go back to bed and that’s literally just from getting up and getting dressed.” (Lauren, F, 36-45)

#### Community, identity and belonging

This theme is concerned with connection and comparison to other people in the development and maintenance of wellbeing. The first subtheme explores receiving an autism diagnosis and its impact on identity, belonging and people’s place in society. The second subtheme explores ways in which people connected with others and achieved a sense of belonging within communities.

##### “Like being let out of prison”: identification with autism diagnosis

Receiving an autism diagnosis, particularly as an adult, contributed to some participants’ sense of identity. Although the experience was initially described by some as a shock, this was followed by relief as it helped people to make sense of their lives, encouraging self-acceptance and understanding. Several people described how their diagnosis relieved longstanding emotional turmoil.

“I used to get very suicidal contemplating ending my life, all the time. [Since diagnosis] that’s all gone - I’ve stopped self-harming, I’ve stopped banging my head, you know it’s, I can’t describe it, it’s like being let out of prison.” (Mandy, F, 56-65)

Identification with their diagnosis (formal or self-identified) increased participants’ understanding of their needs, abilities and disabilities. This helped them develop strategies and make changes to improve wellbeing, including changing their home environments to support their sensory needs, modifying their level of social engagement, and accessing services.

“A parallel I draw is being Asperger (sic.) is a bit like going to a foreign country. […] Their culture is a little bit different, and if you are aware of those differences you can navigate that country a bit easier.” (Norman, M, 66+)

In self-identified individuals, the enhanced self-management was sometimes enough to decide that there was little benefit from pursuing formal diagnosis. Diagnosis and self-identification also improved understanding from others. However, some participants continued to experience shame despite having an increased understanding of their differences.

“I still feel ashamed about it, like really ashamed, that this is how I have to live but I’ve got to a point now where I’ve got, I’ve accepted that that’s the way I’ve got to be in order to stop myself getting overwhelmed” (Lauren, F, 36-45).

##### “You can feel safe and be yourself”: community and belonging

Participants’ experiences of belonging to communities suggested this contributed to wellbeing. Some communities were connected by identity and interests, such as those based around autism or mental health diagnosis, on interests and values such as religious groups and political campaigns, and the LGBTQ + community. These groups provided shared understanding, resources and knowledge, a sense of safety, lack of judgement, and a shared sense of humour.

“There are places that you can feel safe and be yourself, without having to monitor yourself or mask.” (Helen, F, 36-45) [note ‘masking’ in this context refers to autistic camouflaging (see Bradley et al. 2021), not the Covid-19 public health measure].

Many communities had moved online during the pandemic. Although this helped extend their reach, not everyone responded positively to this shift, suggesting it may have lessened community access for some.

“I’ve noticed that some people [from local support group] who were there every time aren’t there on Zoom.” (Norman, M, 66+)

Participants also described barriers to being involved in communities. Social challenges included managing group dynamics and remembering names, difficulty finding supportive communities due to a lack of availability or information, or feeling alienated in some groups. Community events, such as Pride for those who identified as LGBTQ+, could be inaccessible due to crowding and sensory overwhelm. Others felt their identity, interests or values, such as religion, were personal to them and did not want to share them with others.

Many participants felt a lack of closeness and shared values with communities connected by their local area, although others valued their local heritage. Some felt local communities with shared values were reminiscent of the past. Some participants experienced a sense of alienation, hostility and lack of safety in their local area, including crime rates and distrust in local policing. Neighbours often caused problems such as noise, but some also provided support and a social network. Some people had very small support networks of close friends and family members, but valued these. For some participants, a lifelong perception of feeling different from others that impacted on their ability to feel a part of their community.

“I feel very alien, I’m almost convinced I was born on a different planet; I’m like, I’m not human, these people are so different from me.” (Mel, NB, 36-45)

Some people felt problems were due to their difficulties with communication and social interaction. Others felt a lack of understanding and stigma about autism, such as stereotyped media portrayals, was responsible for feelings of alienation.

#### External support and barriers

This theme explores external influences on wellbeing, which may support the person or pose barriers. In the first subtheme, participants described how seeking and receiving support from external sources contributed to wellbeing. In the second subtheme, participants described external influences which impacted negatively on their ability to seek support and manage wellbeing. The third subtheme summarises the positive and negative impacts of the Covid-19 pandemic on wellbeing.

##### “I Find a lot of things by helping other people.” seeking external support with wellbeing

Most participants had accessed professional support services, including psychological and occupational therapies, domiciliary care and support worker assistance. Participants had required help with managing emotions, trauma, relationships, employment and independence. Some participants emphasised the importance of support that was not focused on trying to change who they were as a person, which they felt was tied to being autistic.

“Her normal sort of CBT handbook says do this, do that and the other. And I say hold on, you’re actually really challenging a fundamental part of my persona, you know, this is actually, my autism you’re challenging there and I can’t do this, and she will sort of rethink and take a different approach.” (Ivan, M, 56-65).

Support from the VCSE sector was another route to external support as it was the principal way of accessing autism peer support services, some of which offered unlimited low-cost counselling, which was perceived as easier to access than formal medical pathways. Participants’ wellbeing was also supported informally by friends and family. Friendships were important for managing emotions, sharing advice, enjoying shared interests and reducing loneliness, while family members and partners would help people solve problems, listen to concerns, organise services and manage routines, as well as enjoying leisure time together. However, time away from family was also important for some participants to avoid frustration.

Medication was used by some participants to manage depression, anxiety and ADHD. Finding suitable medication sometimes involved trial-and-error and frequent medication changes due to experiencing side effects or building tolerance. Some participants used technology to manage wellbeing, such as apps which helped build motivation to maintain daily routines, track mood changes to identify triggers and manage executive functioning in tasks such as shopping. A flexible and supportive working environment was also important for wellbeing – this included helpful colleagues, home working and time off for medical appointments.

These external sources of support promoted health, developed skills to achieve goals such as employment and independence, provided structure and routine, and helped with reframing and re-establishing a sense of identity. Some participants also supported others, for example using their skills to provide practical help for friends, volunteering, and facilitating autism support groups, which could lead to discovering further resources to support their own wellbeing.

“I find a lot of things [for myself] by helping other people.” (Bruce, M, 56-65)

##### “They’re still using the medical model”: external barriers to wellbeing

Participants’ accounts suggested that despite benefits of support, there remained persistent barriers to accessing or benefiting from this. Individual barriers included financial difficulty, a lack of motivation or belief in the approach, or a lack of success from support they had received. Some perceived common psychological therapies, such as cognitive behavioural therapy and mindfulness, to be incompatible with their cognitive differences. Furthermore, participants identified gaps in services for autistic adults without learning disabilities. Many desired more follow-on support from a late autism diagnosis, which could be a confusing time. Some participants were given information on autism post-diagnosis, but felt it was not relevant to them and did not help signpost them towards support.

“It was actually quite hard to find […] I was diagnosed about two years before I even started going, and I was like ‘why didn’t I go sooner?’, but it’s just because I didn’t know about it” (Sian, F, 26-35)

Some services they valued had also had reductions in funding, leading to closure. Many participants felt the health service prioritised physical health over mental health, and that the medical approach of services could be inconsistent with their constructs around wellbeing and neurodiversity. Participants felt the lack of research on autistic adults was a barrier to support being made available.

“[Some services are] still using the medical model where you’ve got to change the autistic person and not change the environment to make society more inclusive” (George, M, 66+)

Some environments posed barriers to wellbeing, including stressful workplaces and crowded or noisy environments that caused sensory overload. The barriers identified sometimes limited access to services, such as hospitals, or encroached into the home environment, as well as access to wellbeing-enhancing activity; for example, while many participants recognised benefits of natural environments, some were obstructed by issues such as lack of transport and a lack of accessible green spaces in their local area.

##### “I’m always 100 yards behind the starting line”: covid-19 both facilitated and hindered wellbeing

The COVID-19 pandemic, especially lockdown, had had negative effects on wellbeing for many participants, including isolation, anxiety and lack of outdoor access. It also disrupted usual coping strategies and daily routines, impacting socialisation, exercise and travel, and some people experienced a loss of confidence and skills, such as executive function and social skills. It became more difficult to plan for the future and some participants had had to postpone major plans such as moving home. There were other practical barriers to managing wellbeing due to pandemic restrictions.

“I haven’t been coping with my dietary problems because finding food that I want to eat in the house is quite an issue […] it’s been hard at times to go shopping whenever I want” (Ollie, M, 18-25)

Some participants described feeling more vulnerable, including worrying about susceptibility to illness, although masks and vaccinations helped alleviate worries. Many experienced anxiety around adhering to lockdown rules, which were sometimes perceived as confusing and ambiguous.

However, lockdowns had also presented opportunities to improve wellbeing, such as establishing new working styles and routines, and reducing social demands. Many participants noted a positive effect on their mood from reduced noise levels and increased day-to-day predictability. Several participants felt lockdowns did not impact their lifestyles substantially, for example those already working from home or without existing high levels of social support and activities, who were able to maintain their normal routine. Some participants felt more connected to others through shared experiences of the pandemic and through technology, although some felt online interactions were not as fulfilling as in-person.

“It’s not quite as good. It’s quite difficult to concentrate on the, the square, the square of people […] it’s much harder to stay in the room” (Helen, F, 36-45)

As lockdowns eased, some participants felt they were being left behind once again, for example those who had benefited from increased online interactions.

“Coming out of it […] I’m back in that situation where, in a non-autistic, in a majority non-autistic society I’m always starting 100 yards behind the starting line” (George, M, 66+)

Repeated lockdowns also caused strain as some participants struggled to cope with repeated isolation and confusing guidelines.

## Discussion

This study investigated autistic adults’ subjective understandings and perceptions of wellbeing, to establish implications of this for personalised practice models, such as social prescribing. Most participants’ definitions of wellbeing were multidimensional; this mirrors public health frameworks such as Five Ways to Wellbeing (described on the National Health Service [NHS] website) and the Office for National Statistics (2016) definition suggesting that common experiences of wellbeing in the general population also resonate with autistic adults. However, the research also identified how the lived experience of autism contributes to these concepts. Participants had developed strategies for promoting positive wellbeing, through actions, routines, identity and connecting with others. Beliefs about what wellbeing meant in relation to their own lives informed these strategies. The study demonstrated that a positive sense of wellbeing can be achievable for autistic people, a perspective that has sometimes been obscured by mainstream research narratives (Chapman and Carel 2022). However, they also identified barriers, both internal and external, to achieving and maintaining optimal wellbeing.

Research by Kim (2019) has suggested self-determination underlies many findings on quality of life for autistic adults. Similarly, self-determination appears to link the themes identified in the present study. The wellbeing activities described by participants were often intrinsically or internally motivated, for example through enjoyment and a prioritisation of present wellbeing over longer-term health goals. Autonomy is an essential component of intrinsic motivation (Gagné and Deci 2005). Participants demonstrated autonomy by pursuing their interests and developing actions for meeting wellbeing goals, cultivating a positive sense of self and establishing themselves within communities, in ways which aligned with their personal definitions of wellbeing, their identity and values. This reflects the SDT concept of integrated regulation, a type of internal motivation. Other participants developed their wellbeing strategies to achieve a goal or to relieve anxiety, which are closer to the centre of the spectrum of internal and external sources of motivation. Some accounts of wellbeing also suggested participants perceived expectations of others, aligning with the construct of external regulation and showing that many considered wellbeing interpersonal as well as individual. Sometimes, not meeting perceived standards due to poor health or internal barriers including executive dysfunction and low energy, led participants to feel less capable of achieving good wellbeing. In the case of those who lacked motivation to maintain their wellbeing in the way they wanted, this was sometimes due to a lack of external regulation, such as not having another person to exercise with.

One finding differing from previous research on autistic adults’ wellbeing was a narrative suggesting external support was a facilitator to wellbeing through enabling independence and development of skills to support wellbeing self-management. Although a lack of need for support has been interpreted by participants and researchers as a marker of autonomy and success (Webster and Garvis 2020), it could be argued that choice to seek and choose support is an expression of autonomy for those who face more profound barriers and reliance on support should not be considered a poor outcome in itself. The gains in health, employment, independence and motivation may facilitate self-determination. However, the results showed that to achieve a benefit to wellbeing it was important supports were accessible, relevant and easy to navigate, which was not always the case.

Connecting with others was also a recurring theme; despite social and communication challenges, many participants were motivated to establish and maintain social connections, which was achieved through seeking or encountering others connected by interest or identity. Belonging within communities encouraged a sense of safety, shared identity and positive roles. Participants also described not having to mask their autistic traits, which, despite short-term gains for social acceptance, has a negative long-term association with wellbeing (Bradley et al. 2021; Cassidy et al. 2018). However, outside of specialist groups participants sometimes felt disconnected from others. Along with other studies that have challenged normative biases in autism research (e.g. Cassidy et al. 2018; Welch et al. 2019; Mitchell, Cassidy, and Sheppard 2019), our findings contrast to theories which suggest autistic people lack social motivation (Chevallier et al. 2012). Instead, sensory overwhelm, miscommunication and social exclusion were barriers to social connection.

Some participants’ experiences suggested some positive aspects of wellbeing were related to being autistic. For example, having focused intrinsic interests presented people with opportunities for employment, occupation and connection with others, and were seldom described negatively with regard to wellbeing, reflecting previous findings (Koenig and Williams 2017). Autistic adults’ motivation for engaging with interests is generally intrinsically motivated and integrated with personal values, a sense of enjoyment and flow, compared to more externally driven motivations (Grove et al. 2018; Grove, Roth, and Hoekstra 2016). Participants’ accounts in the present study also demonstrated flow, a state of absorption and competence around an activity (Csikszentmihalyi 1990). Competence is one of three psychological needs outlined as underlying intrinsic motivation in SDT, along with autonomy and connection (Gagné and Deci 2005). Previous research has found autistic people, their parents and employers note strengths including attention to detail, focus and creativity (Cheriyan et al. 2021; Russell et al. 2019; Scott et al. 2017; Warren et al. 2021); these attributes were also reflected within these accounts. However, the enjoyment of interests suggested positive experiences of autism do not have to be tied to productive value to be seen as worthwhile. While these experiences may also reflect interests in the wider population, for autistic people these are considered a restrictive behaviour, sometimes targeted through treatment. However, scholars with lived experience of autism have proposed that monotropism, a neurological processing style, underlies differences in focus, executive function, sensory and social processing that may compel an enhanced focused on interests, within a spectrum of attention that spans the wider population (Murray, Lesser, and Lawson 2005).

Receiving an autism diagnosis also positively impacted wellbeing by making sense of past experiences, which encouraged self-acceptance. Autistic people often construe autism as an integral part of identity (Botha, Dibb, and Frost 2022) and diagnosis can aid self-understanding and encourage empowerment and autonomy to restructure identity (Lilley et al. 2022). However, stigma about autism and others’ negative responses to disclosure can also link an autism diagnosis with reduced wellbeing (Botha, Dibb, and Frost 2022; Chapman and Carel 2022; Lilley et al. 2022). Several participants in the present sample described seeking an autism diagnosis late in life; although some had already developed adaptive strategies, for others diagnosis was important for making changes to support wellbeing. Although it sometimes evoked negative emotions, another study found this often dissipates over time as a more positive identity develops (Corden, Brewer, and Cage 2021). This may lead to improved wellbeing and reduced stigma (Gillespie-Lynch et al. 2017; Maitland et al. 2021). This demonstrates how receiving an accurate and affirmative diagnosis at any age may support autonomy and wellbeing.

However, some participants experienced disabling internal barriers to wellbeing including executive dysfunction, burnout, fatigue and co-occurring physical and mental health conditions. Furthermore, although participants’ wellbeing management strategies demonstrated autonomy, without the capability for resilience and flexibility some routines became restrictive, negatively impacting on wellbeing if these were disrupted. Environments and their interaction with sensory processing were other contributors to wellbeing, although a minority of participants did not regard these aspects as important. Environmental barriers, including the COVID-19 pandemic, sometimes led to a need for additional support from external sources, and reduced autonomy and competence. However, new opportunities presented by a changing environment may have renewed a sense of autonomy that helped some people achieve wellbeing goals in novel ways, though during the pandemic the isolation and anxiety experienced in lockdown reduced wellbeing for others. These findings add to research showing mixed impacts of the COVID-19 pandemic on autistic people’s wellbeing (Bundy et al. 2022; Mosquera et al. 2021; Pais and Knapp 2021). The person-environment interaction can be overlooked by studies which focus on individual traits as markers of wellbeing, yet Lai et al. (2020) emphasise person-environment fit as a key component of support for autistic people at all stages of life. To maximise benefits of approaches such as social prescribing it will be especially important to support navigation of barriers, modifying environments in which activities take place and manage executive dysfunction and burnout.

### Application to practice: social prescribing

The approach of social prescribing may align with our participants’ views of wellbeing as holistic and multidimensional by allowing people to focus on areas of wellbeing which matter to them; some participants were wary of more medicalised approaches. Social prescribing may be able to support wellbeing for autistic people through signposting to activities that build on intrinsic interests and strengths, which could create opportunities for enjoyment, flow, connection with others and self-development. Volunteering, for example, could connect people with meaningful activity in areas of interest.

Research has found that peer support groups can benefit autistic adults through empathetic interactions and an accepting social environment, which help to build positive self-understanding and resilience (Crane et al. 2021; Crompton et al. 2021). On the other hand, quantitative research is yet to demonstrate strong evidence for the impact of peer support on mental wellbeing in general samples (Blodgett, Kaushal, and Harkness 2022), however the present study and previous research suggests that for autistic people, social participation is an important implicating factor for wellbeing and self-determination (Kim 2019). As peer support opportunities were sometimes difficult to find, social prescribers could facilitate this by signposting to local community autism groups and charities, especially those led by autistic adults.

External supports also included self-directed use of technology, which aided motivation, executive function and social connection, especially in the context of COVID-19. Historically, technology has been fundamental in connecting autistic communities previously experiencing isolation and lacking a collective voice (Bagatell 2010). Recently, research has shown that autistic adults appreciate technology which “scaffolds” independence rather than aiming to reduce autistic traits (Zheng et al. 2022), reflecting the motivations of seeking support in the present sample. Social prescribing approaches could signpost to digital solutions such as apps to support wellbeing. Although access to other resources such as books, education and information are common forms of social prescription (Chatterjee et al. 2018), the potential of prescribing technology to enhance wellbeing has not been widely discussed. Finally, it is important to note that although autistic adults struggling with wellbeing may require intervention, not all autistic adults will need or want support at all times. Some participants identified a desire to access inexpensive equipment to support their wellbeing; schemes such as enablement funds may facilitate independence for individuals requiring less direct support.

### Strengths and limitations

A strength of this study is its diverse sample, comprising people of a range of ages, genders, occupations and life experiences, as well as those who self-reported having learning disabilities, physical comorbidities, and difficulties communicating through speech. The findings suggest it could be worthwhile for future research using quantitative or mixed methods designs to explore how age, occupation, relationships and intersectional aspects of identity, such as gender, may be associated with wellbeing for autistic adults; these were not identified as broad themes within the data but were relevant to some participants’ experiences. In particular, research on autistic older adults is very limited (Michael 2016); the higher proportion of participants in older age bands may help to highlight wellbeing issues applicable to an older population, such as the impact of receiving a diagnosis late in life.

Remote interviewing increased reach to a wider pool of participants and during tiered Covid restrictions was essential for this study, but may be less accessible for some with higher communication needs, who were less represented by this study. Alternative methodologies, such as PhotoVoice, may be more suitable for capturing understandings of wellbeing in autistic samples with these needs (e.g. Lam et al. 2020). The use of critical realism strengthened the study by enabling the identification of internal barriers to wellbeing but situating these within the wider social context, such as the pandemic, neither positioning autism as inherently detrimental to wellbeing (in many cases, participants identified positive experiences associated with autism) nor dismissing disabling experiences. This provides justification for personalised care models focused on autonomy over more standardised approaches.

Participants were also invited to choose a topic to begin the interview, which shifts control from the research team and led to some important insights not identified by the interview schedule such as the impact of adult autism diagnosis on wellbeing. However, by using the methodology of semi-structured interviews, this study may still focus too strongly on wellbeing as an individualised concept. Future research could explore autistic adults’ wellbeing on a collective and societal level, such as further research into ethnographies of autistic community wellbeing, research into which is in its infancy. For example, Idriss (2020) used ethnographic methods to explore autistic social infrastructure and community strengths.

## Conclusions

This study affirms that wellbeing and autonomy are achievable for autistic people and that while these can be linked to the experience of being autistic, there is much common ground in wellbeing constructs relevant to autistic adults and the general population, suggesting that generic theories and supports relating to wellbeing may be inclusive of this population. For example, self-determination theory may be an avenue for further theoretical exploration in relation to autistic adults’ wellbeing as a persistent thread throughout the identified themes. This suggests that costly autism-specific services may not always be necessary to support and promote wellbeing. Social prescribing, as an example, could foster self-determination for autistic adults in a similar way to other populations, by facilitating navigation of barriers and working alongside individuals to identify existing strengths, supports and opportunities to promote wellbeing self-management, connection to communities and autonomy.

## Appendix 1

### Interview schedule: questions on wellbeing

Are there any thoughts you’ve had about this topic of managing your health and wellbeing that you would like to start off with?

What does wellbeing mean to you?

- What does it mean to feel at your best? When was the last time you felt that way? What helped you feel like that?
- What do you do to keep mentally and physically healthy? Is this important to you? How did you develop these strategies? Can you think of a time when they were especially helpful? How about a time they have not been helpful?
- Have there been any differences in how you are able to keep well during the Covid pandemic compared to before?

How do different places and environments around you affect your wellbeing?

- Where do you feel at your best? What aspects of that place do you think help with this?
- Are there any environments that you don’t like? How do these make you feel? Are there any aspects you’re able to control to try to improve the experience? What parts are out of your control?
- How do you feel about nature and green spaces? What types of natural spaces do you like most? What features of a natural space would not appeal to you? How do you feel when you spend time in nature? Why? How important is nature to you as a person?
- How do you feel about the local green spaces where you live? How important are these spaces to you in your life? Would anything help you to use green spaces in your local area more?
- How has COVID-19 affected how you experience different places and environments? Has anything about the experience changed?

How do you feel about the local community in your area?

- Do you feel a part of that community? What makes/would make you feel part of your community?
- Is anything missing from the experience?
- Can you give me an example of a community you feel that you a part of?
- How would you define a community?
- How has COVID-19 affected your experience of belonging to communities? What impact has this had on your life?

Is there anything else you’d like add about these topics? Is there anything that we’ve discussed today that you feel would be the most important area for research to address?
